# Perceptions of first-degree relatives of patients with rheumatoid arthritis about lifestyle modifications and pharmacological interventions to reduce the risk of rheumatoid arthritis development: a qualitative interview study

**DOI:** 10.1186/s41927-018-0038-3

**Published:** 2018-11-06

**Authors:** Gwenda Simons, Rebecca J Stack, Michaela Stoffer-Marx, Matthias Englbrecht, Erika Mosor, Christopher D Buckley, Kanta Kumar, Mats Hansson, Axel Hueber, Tanja Stamm, Marie Falahee, Karim Raza

**Affiliations:** 1 Institute for Inflammation and Aging, Rheumatology Research Group, College of Medical and Dental Sciences, University of Birmingham, Birmingham, B15 2TT UK; 2 Department of Psychology, Nottingham Trent University, 50 Shakespeare St, Nottingham, NG1 4FQ UK; 3 Section for Outcomes Research, Center for Medical Statistics, Informatics, and Intelligent Systems, Medical University of Vienna, Spitalgasse 23, BT88/E 031090 Vienna, Austria; 4 University of Applied Sciences FH Campus Wien, Vienna, 1100 Austria; 5 Department of Internal Medicine 3, University of Erlangen-Nuremberg, Internistisches Zentrum (INZ), Ulmenweg 18, 91054 Erlangen, Germany; 6 Division of Rheumatology, Department of Internal Medicine III, Medical University of Vienna, Vienna, Austria; 7 Department of Rheumatology, Sandwell & West Birmingham Hospitals NHS Trust, Birmingham, UK; 8 Arthritis Research UK Rheumatoid Arthritis Pathogenesis Centre of Excellence, MRC Arthritis Research UK Centre for Musculoskeletal Ageing Research and NIHR Biomedical Research Centre, College of Medical and Dental Sciences, University of Birmingham, Birmingham, UK; 9 The Institute of Clinical Sciences, College of Medical and Dental Sciences, University of Birmingham, Birmingham, B15 2TT UK; 10 Faculty of Biology, Medicine and Health, School of Health Sciences, University of Manchester, Manchester, M13 9PL UK; 11 Centre for Research Ethics and Bioethics, Uppsala University, Box 564, SE-751 22 Uppsala, Sweden

**Keywords:** Rheumatoid arthritis, Risk, Qualitative, Relatives, Preventive medicine, Lifestyle changes

## Abstract

**Background:**

There is increasing interest in the identification of people at risk of rheumatoid arthritis (RA) to monitor the emergence of early symptoms (and thus allow early therapy), offer lifestyle advice to reduce the impact of environmental risk factors and potentially offer preventive pharmacological treatment for those at high risk. Close biological relatives of people with RA are at an increased risk of developing RA and are therefore potential candidates for research studies, screening initiatives and preventive interventions. To ensure the success of approaches of this kind, a greater understanding of the perceptions of this group relating to preventive measures is needed.

**Methods:**

Twenty-four first-degree relatives of patients with an existing diagnosis of RA from the UK, three from Germany and seven from Austria (age: 21–67 years) took part in semi-structured interviews exploring their perceptions of RA risk, preventive medicine and lifestyle changes to reduce RA risk. Interviews were audio-recorded, transcribed verbatim and analysed using thematic analysis.

**Results:**

Many first-degree relatives indicated that they anticipated being happy to make lifestyle changes such as losing weight or changing their diet to modify their risk of developing RA. Participants further indicated that in order to make any lifestyle changes it would be useful to know their personal risk of developing RA. Others implied they would not contemplate making lifestyle changes, including stopping smoking, unless this would significantly reduce or eliminate their risk of developing RA. Many first-degree relatives had more negative perceptions about taking preventive medication to reduce their risk of RA, and listed concerns about potential side effects as one of the reasons for not wanting to take preventive medicines. Others would be more willing to consider drug interventions although some indicated that they would wish to wait until symptoms developed.

**Conclusions:**

Information targeted at those considered to be at risk of RA should contain information about RA, the extent to which risk can be quantified at an individual level and how risk levels may differ depending on whether early symptoms are present. The benefits (and risks) of lifestyle changes and pharmacological interventions as potential preventive measures should be clearly described.

## Background

Rheumatoid arthritis (RA) is a common chronic inflammatory disease with a prevalence of 1% [1]. The disease has a significant negative impact at both individual and societal levels [2]. Early diagnosis and treatment reduce the risk of future joint damage and disability [3–8] and increase the chance of drug-free remission [9]. As a result, there is now a drive to identify and treat people as early in the disease process as possible, potentially before the onset of joint swelling [10].

Genetic factors contribute significantly to a person’s risk of developing RA [11]. Having a family history, especially having one or more first-degree relatives with RA, increases the risk of developing RA by approximately 3–5 fold [12–14]. Modifiable environmental factors such as smoking [15], alcohol intake [16, 17], and diet [18–20] also influence the risk of developing RA [21]. Interactions between these risk factors are likely [22]. Gathering information about an individual’s genotype, environmental exposures, systemic and joint related immune abnormalities may thus allow clinicians to predict future RA development in those who are currently asymptomatic [22]. Among those people with a close relative with RA, current risk models using both environmental and genetic factors have been shown to be highly discriminative for both seropositive and seronegative RA [23].

Relatives of RA patients are a prime target population for both risk stratification and preventive interventions [24, 25]. First-degree relatives of RA patients are currently in the focus of prospective observational cohort studies [26–28] and interventional trials [21, 25]. However, difficulties recruiting first-degree relatives to such studies have been reported [29]. As recruitment of first-degree relatives is usually dependent on the cooperation of RA patients themselves, it is also important to understand patients’ perceptions of RA risk, risk modification and the communication about these issues with their relatives. Qualitative studies have already started to explore the views of RA patients in this context [30].

In order to develop effective recruitment strategies and potential future preventive strategies for first-degree relatives we also need to have a good understanding of the perceptions this group, and their willingness to engage with preventive approaches. We have recently reported data from interviews with first-degree relatives in three European countries looking at their perceptions of being at risk of RA and of predictive testing. Relatives reported having  a range of concerns about both predictive testing and risk information related to RA, including the possibility that knowing ones risk might increase anxiety and reported concerns about the level of uncertainty associated with predictive testing. Those relatives who expressed positive views about predictive testing indicated that they would need support to understand risk information [22].

Recent qualitative research has suggested that uptake of preventive medication by first-degree relatives might be related to perceived baseline risk and experience of the disease through their relative(s) [31]. Furthermore, preliminary data from a choice experiment with first-degree relatives suggest that acceptance of such medication would depend on its effectiveness and side effect profile [24]. In relation to lifestyle changes, one recent trial looked at the effect of disclosure of first-degree relatives’ RA risk personalised with genetics, biomarkers, and lifestyle factors on their health behaviour intentions and actual behaviour [21] and found that those at risk were motivated to and actually changed certain behaviours after such personalised information. However, there is currently no information available about first-degree relatives’ perceptions around these lifestyle changes and pharmacological interventions aimed at reducing the risk of RA development. This paper provides an exploration of previously unreported data collected during our earlier interview study [22] with first-degree relatives examining these issues in more detail.

## Methods

### Participants

Eligible participants were the first-degree relatives of people with an existing diagnosis of RA. Patients with RA were approached during their routine secondary care clinic appointments in Birmingham (United Kingdom), Erlangen (Germany) and Vienna (Austria) and asked to consider contacting a first-degree relative about participation in an interview study about risk and predictive testing for RA. Full procedural details are presented elsewhere [22]. We restricted the selection of relatives to offspring and siblings and excluded parents. In order to be eligible to take part, first-degree relatives had to be aged 18 years or over and not diagnosed with inflammatory arthritis at the time of the study.

### Data collection and analysis

The semi-structured interviews were conducted either face to face at the recruiting hospital sites or by telephone and were guided by an interview schedule (Table 1). The schedule was informed by a literature review [32, 33] and in consultation with an international team of healthcare professionals, researchers and patient research partners participating in the EuroTEAM (Towards Early diagnosis and biomarker validation in Arthritis Management) project [34]. The interviewers in the UK were conducted by RS (female research fellow with a psychology background) and KK (female researcher and nurse specialist)). Interviews in Germany were conducted by AH (male senior clinical research fellow) and interviews in Austria were conducted by EM (female occupational therapist and researcher). All interviewers had previous experience of conducting interviews and RS, KK, and EM have extensive experience of qualitative methods. Interviewees received information about EuroTEAM in the participant information sheet (i.e. that it is a multi-country research project funded by the EU) and were told the name of the person interviewing them, as well as their role in the project. All interviews were audio recorded and transcribed by a professional transcription service. Interviews conducted in Germany and Austria were translated from German into English following transcription by bilingual native speakers and the translations were checked by the respective research teams. All transcripts were analysed centrally at the University of Birmingham, United Kingdom.

**Table 1:** Outline Interview schedule

• Tell me what you know about RA?	
• Do you ever worry about the possibility of developing RA in the future?	
• What would you think if you were told that you could have a test that would tell you how likely you were to develop RA?	
• What would your concerns be if you knew what your risk of developing RA was?	
• What kind of tests do you think people might be able to do to work out whether or not you might develop RA (tests that are available now and tests that might become available in the future)?	
• Various tests can currently be done, and various tests are currently being developed to predict the development of RA. What are your thoughts about: ° Blood tests looking at biomarkers, molecules in the blood ° Blood tests looking at genes ° Tests involving scanning the joints with either an ultrasound or MRI ° Tests involving taking tissue out of a joint or elsewhere (e.g. lymph nodes)	
• What are your thoughts about taking medicines to reduce the risk of RA developing in the future?	
• What are your thoughts about changing your lifestyle (e.g. stop smoking, more exercise, change diet) to reduce the risk of developing RA in the future?	

Data collection and analysis were carried out in parallel to allow for an assessment of when thematic saturation had been achieved. Transcripts were analysed thematically [35] using NVivo (software programme for qualitative data analysis; [36]). One researcher (RJS) coded all the transcripts and three patient research partners blind coded three transcripts. The coding framework was discussed with the patient research partners and coding categories that lacked concordance were absorbed into the coding framework. The initial codes were then grouped into the most noteworthy and frequently occurring categories by RJS, KK and GS.

## Results

Thirty-four first-degree relatives of RA patients were interviewed; 24 from the UK, three from Germany and seven from Austria. Participants were aged between 21 and 67 years (*M* = 39, *SD* = 10.8), and 76% (*n* = 26) were female. All but two British participants were white. Table 2 gives the individual participant characteristics including age, gender, relation to relative with RA and current musculoskeletal symptoms.

**Table 2:** Interviewee characteristics

Participant number	Gender	Age	Ethnicity	Interview Country	First degree relative with RA	Experience of (blood) testing^a^	Reported musculoskeletal problems^a^
1	Female	18–40	White	UK	Parent	None	None
2	Female	41–60	White	UK	Parent	None	Yes (Historic)
3	Male	61–80	White	UK	Sibling	None	None
4	Male	18–40	White	UK	Parent	None	None
5	Female	18–40	White	UK	Parent	None	None
6	Male	18–40	White	UK	Parent	Yes	None
7	Female	18–40	White	UK	Parent	None	Yes
8	Female	18–40	White	UK	Parent	Yes	Yes
9	Female	41–60	White	UK	Sibling	None	Yes
10	Female	18–40	White	UK	Parent	None	Yes
11	Female	41–60	White	UK	Sibling/Parent	None	Yes
12	Female	41–60	White	UK	Sibling	None	Yes
13	Female	41–60	White	UK	Sibling/Parent	Yes	Yes
14	Female	41–60	White	UK	Parent	Yes	Yes
15	Female	18–40	White	UK	Parent	None	None
16	Female	18–40	White	UK	Parent	None	None
17	Female	41–60	Asian	UK	Parent	None	None
18	Female	18–40	White	UK	Parent	None	None
19	Male	41–60	Asian	UK	Parent	None	None
20	Female	18–40	White	UK	Parent	None	None
21	Female	41–60	White	UK	Parent	None	Yes (Historic)
22	Female	18–40	White	UK	Sibling	None	None
23	Female	41–60	White	UK	Parent	None	None
24	Male	41–60	White	UK	Parent	None	None
25	Female	18–40	White	Germany	Parent	None	None
26	Female	18–40	White	Germany	Parent	None	None
27	Female	41–60	White	Germany	Parent	None	None
28	Female	18–40	White	Austria	Parent	None	None
29	Male	18–40	White	Austria	Parent	None	None
30	Female	61–80	White	Austria	Sibling	None	None
31	Female	18–40	White	Austria	Sibling	Yes	None
32	Male	18–40	White	Austria	Parent	None	None
33	Male	18–40	White	Austria	Parent	None	None
34	Female	18–40	White	Austria	Parent	None	None

^a^Data on testing and musculoskeletal problems are based on self-reports of the interviewees as interviewers had no access to health records of the interviewees

Interviews lasted between 30 and 90 min. The results presented here represent a new analysis of the resulting transcribed data. Findings related to an analysis of perceptions of risk and predictive testing are presented elsewhere [22]. The core themes presented here focus on perceptions of first-degree relatives about the possibility of making lifestyle changes and taking preventive medicine to modify their risk of developing RA. An overview of the organising themes and subthemes can be found in Table 3. Quotations referred to in the text can be found in Tables 4 and 5.

**Table 3:** Overview of themes related to the sub-analysis focused on modifying risk through lifestyle and preventive medicines

Modifying risk through lifestyle intervention	
• Positive view of lifestyle changes and/or continuing to engage with healthy living to reduce risk of developing RA	
• Healthy eating, diet and exercise as examples of life style changes	
• Being overweight considered a risk factor	
• Knowing risk is useful as it allows you to make life style changes as a preventive measure	
• Need for more information about effectiveness in order to make a decision about lifestyle changes	
• Perceived negative consequences of making life style changes	
• Unwilling to make lifestyle changes including smoking cessation, unless it is clear that there will be a significant reduction in risk	
Willingness to take preventive medicines to modify risk	
• Uncertainty and worry about potential short term and long term side effects	
• Perceived need to consider pros and cons carefully	
• Weighing perceived uncertainty of developing RA against perceived certainty of side effects	
• Level of likelihood of getting RA affects consideration of preventive medicine	
• Negative opinion about taking medicines in general	
• Preference for making lifestyle changes over taking preventive medication	
• Recognition why medication might be used	
• Preference for starting medication only when first symptoms appear	
• Screening will put at risk individuals on alert for early symptoms	
• Perceived effectiveness of intervention (medication or lifestyle changes) makes a significant impact on acceptability

**Table 4:** Modifying risk through lifestyle changes

*1*	*“Well, I would gather information ahead of time about this disease, so, what issues are there, what characteristics, and how can you get rid of it, how can you prevent it so that it doesn’t break out. Well, I would be more careful about my life or my health than I have up to now.” (Participant 33; male)*
*2*	*“…..it would strike a chord with me to eat healthier and be healthier if they said I can have five years extra when I’m possibly not going to get it. I’d rather not have RA than have it and I’d do everything in my power not to have it because it doesn’t seem that much fun.”* (Participant 5; female)
*3*	*“Well, I don’t smoke and I hope I’ve got a reasonably healthy diet. Yes, if there are changes necessary to be made, I’d be happy to make them.”* (Participant 3; male)
*4*	*“Lifestyle changes, I’m up for any kind really, yeah. Healthy eating and exercise, although I can’t do a lot but I do try and do as much as I can.”* (Participant 13; female)
*5*	*“How I look at it is, it’s part of… if you get it the only way that you can actually do something about it is change … what you eat, the foods you consume and stuff like that. So I’m the only one in my family that eats organic or free range food and I drink whole milk and I eat saturated fat as well so I avoid low fat stuff so I believe it’s to do with your diet as well. By changing your diet you can actually change the way your body functions and what happens to it.”* (Participant 14; female).
*6*	*“I think it probably half depend on what kind of person you are, I know for my sister she was much more worried than I was only because she’s a lot older than me and she’s overweight and she saw that as kind of, like without reading the letters I could figure she was going to get it more than me.”* (Participant 5; female)
*7*	*“I would prefer to know about* (my risk of developing RA) *because then I can potentially try and manage my lifestyle better in general ..…”* (Participant 24; male)
*8*	*“So,… lifestyle changes, yeah definitely, particularly with things like exercise, because that’s something, I’m not a particularly sporty person, and I know my dad’s been very, very sporty throughout his life and, you know, what are the risks I suppose, of that? Or whether* (it is) *osteoarthritis that puts the weight on your joints or, you know, that kind of information that I wouldn’t necessarily know; that’d be the kind of thing I’d be looking to find out.”* (Participant 1; female)
*9*	*“I think that I might behave more consciously in ways of diet or, yes generally, maybe I would try somehow to prevent that it actually happens. Well, of course, I would have to read up on what is possible in that regard, but I think I would do that.”* (Participant 31; female)
*10*	*“Well, I wouldn’t change anything about my diet and concerning sports, as I said, that’s the question, I don’t think so, due to the fact that I think it wouldn’t stress me, I wouldn’t change my habits in sports as in doing less or doing more, whatever, because I just think, then I would become so careful that in the end I would just sit around and not do anything anymore or exaggerate everything. That wouldn’t be a good idea, well I can’t really imagine that, because basically I would change little or nothing.”* (Participant 28; female)
*11*	*“If all I know that I am at a heighted risk, I don’t think I would change my diet”* (Participant 28; female)
*12*	*“Yeah, I think it would have, because after seeing what my mum can go through, when it does trigger and it kicks in, yeah, without a doubt, I think, it brings reality home. So, yeah, I think if, I’d have probably walked out and chucked the fags in the bin there and then if, certainly if they’d come back and said, ‘Yeah, you’re in this category,’ and I thought, certainly if it was in, probably, middle to upwards, I definitely would have. If it had been the other way, I’d have thought, ‘Right, I need to quit,’ and worked on a couple of months’ timescale to do it.”* (Participant 6; male)
*13*	*“I’ve been smoking since I was 14. I’m 31 now, so I’m thinking, ‘Well, I’ve been smoking longer than half of my life.’ I wanna be around for my son when he’s older, … I know smoking is – because unfortunately I am a smoker – and my mum was, like, ‘You need to pack it in, anyway, but even more so after it had ...’ Certain things can trigger it …Yeah. I’m trying to quit smoking anyway, at the moment, so I’ve reduced the fags down to four at the moment, so I’m working down to getting to nothing.”* (Participant 6; male)
*14*	*“Yes, that’s a very good question. Since I’m a smoker and to be honest my diet isn’t really that great, I don’t know if I’d really – if someone could confirm to me, 100%, that if I stopped smoking and had a healthier diet I could stop myself from developing RA, then I would consider it – I would try to stop smoking and live a healthier life.”* (Participant 26; female)
*15*	*“Regarding smoking, I mean I smoke, I wouldn’t know, well if I say I have a heightened risk and that chances are 50:50 I develop it or not, then I don’t know whether I would consider it worth it to stop smoking.”* (Participant 28; female)
*16*	*“… ..People can be very touchy, particularly about smoking. About dietary things, people are more willing to take on board the advice about leafy green vegetables and those kind of things, but not smoking. I think there’s a little bit of them that makes them a bit over-defensive about their choice. They get hammered from so many different aspects; I think that it’s, like, one more person, that they don’t wanna be told from another person, that they shouldn’t smoke, so it’s quite a delicate subject. You can, sort of, give them the facts without trying to scare them, I guess.”* (Participant 1; female)
*17*	*“I’d be happy to change my lifestyle, if I thought it was going to have an impact on me developing the disease. But like you say, the only real strong lifestyle – well, the only one that I’m really aware of that has a massive impact, is smoking. I don’t smoke. You can’t really help but come into contact with people that do smoke, as much as you try and limit it, and it’s not that much now.”* (Participant 10; female)

**Table 5:** Modifying risk through preventive medicine

1	*“For me, it would depend, if I knew for sure that the danger of my developing it within the next 5 years was very high, then I would definitely try to find out what kind of side effects it has. Most medicine has side effects and in the long run that might not be so good”.* (Participant 31; female)
2	*“I would definitely want to find out more about taking the medication. I do worry about the side-effects of some of the drugs. I’d have to take that into account as well. Mild side-effects would be fine. If it was something that affected my everyday life in a negative way now then I would have to weigh-up the pros and cons, even if it was worthwhile in the future. I’d want to know the side-effects, or the possible side-effects. Maybe even give it a trial run. Maybe I would just do that. Try it out and see because it affects people in different ways, doesn’t it? It’s not guaranteed that you would get all of the side-effects.”* (Participant 20; female)
3	*“I do think sometimes prevention is better than a cure but when I say prevention’s better than a cure I mean sometimes you know just by looking after yourself and things like that. Whether I could take medication for something that’s not there I’m not sure about because I would presume, I’d have to know a bit about the medication, would it be some sort of steroid you know because some medications can affect you in other ways so I would have to know what it was and what my effects would be before I would consider something like that.”* (Participant 21; female)
4	*“Side effects. I would be concerned about side effects. I know with a lot of medicines you get a lot of side effects. You’re taking the medication for one thing and then you’ve got something else developing or something else coming out of it. And being a diabetic and suffer with diabetes, blood pressure as well, and the digestive system. So I’m a bit worried about side effects that the medication would have.”* (Participant 13; female)
5	*“I don’t know really. I’d have to try it but then I’d probably read up more about it, what the side effects are, because if they’ll clash with my other medication, because if they did then the side effects I’d probably just leave it as it is then if there’s going to be worse side - if I’m going to be feeling worse than I already am or with the pain. And then maybe it all depends. I might just stick to the painkillers and try to avoid the pain. I really don’t want too many side effects.”* (Participant 13; female)
6	*“If I had to start taking that medication would that affect me having kids.”* (Participant 5; female)
7	*“They’ve got side effects, because I know that my mum came off the methotrexate because I think, she’d got some – I think on the x-rays of her lungs and that, there was some shadowing, so I think he took her off the methotrexate because of that.”* (Participant 2; female)
8	*“So, unless you can tell me it’s going to be a significant benefit at preventing onset of disease, again, I wouldn’t want to take any medication unless it was going to really significantly reduce my chances of developing it. So it would depend on the sort of risk … benefit profile of the two.”* (Participant 10; female)
9	*“I would be disinclined to take any medicines purely based on a probability factor. I would want more definitive evidence before I’d start taking medicines.”* (Participant 4; male)
10	*“I’ve got to take a medication for how long, the rest of my life? … It’s a big commitment when the odds of developing the disease is still fairly high if I’ve got a 50% risk of still developing it, whereas if you tell me, ‘Well, actually, if you take it and based on what we can tell you about your predictability factors, your odds of developing the disease are gonna be down to 5%,’ then I might consider it..”* (Participant 10; female).
11	*“But it’s the weighing up, do I want to take a medication for the rest of my life, potentially, if it’s preventative? What happens if you stop it? Again, you need trials to go on a long, long time to tell us that, and you’re just not gonna have that data … you know, even once you’ve got your predictive things, you’re not gonna know how long somebody has to stay on that medication to prevent it. So it’s a bit commitment for the rest of your life, to stay on a medication.”* (Participant 10; female).
12	*“Okay, so I think if it’s a number, I think it’d have to be fairly high for me to wanna take a preventative tablet. So probably that’d be something I think I’d give some thought, I guess, but I suppose 50% probably wouldn’t be high enough for me to want to …do preventative medicine, but obviously I’d be looking out for symptoms to start, you know … medication when I developed symptoms. But, no, if it was as high as, sort of 70%, 80%, I probably be more likely to say, Okay, let’s do the preventative.”* (Participant 01; female)
13	*“Nothing, I don’t like pills I don’t want to say, but I don’t think much of taking medicine, I’ve always been someone who didn’t like taking medicine, in that case* (taking medicine to reduce the risk of developing the disease) *I would say no”* (Participant 32; male).
14	*“Just I never liked taking medication. Only if I have to I would take it. If I can get away with not taking it I will not take it. I have done it in the past. Get away with things. I’ve been told I’ve got something, not took medicine for it. They did a lot of trying to persuade me and I said no. I was adamant that I hadn’t got that, which I was right. So I didn’t take the medication and 12 months they said, ‘Right, okay, you’re clear.’ They kept prescribing me the medication but I wouldn’t take it.”* (Participant 13; female)
15	*“I try to exercise. So, I don’t know that I could modify my lifestyle, but if it was a choice – if I had options to changing my lifestyle, and it was a choice between that and the long-term medication, and the benefits were equal between the two, I’d go for lifestyle over a medication.”* (Participant 10; female)
16	*“Yeah, I think* (with) *that kind of information, then, I’d be much more keen to, sort of, sort out what I needed to do to try and prevent that becoming a problem; if I could at that point take some medication to, sort of, reduce the antibodies or you know, head it of before it became a big problem.”* (Participant 2; female, 42)
17	*“Dad’s been pretty bad with it at times and fortunately, at the moment, he seems to be on a fairly even keel, which is good. So I think having seen at first- hand, I’d be much more willing to ... And then given that, if my testing came back and said I’d had a high risk, I’d be much more willing to, sort of, then consider medication - particularly if it was* (going to be) *affecting my arms - a mild tablet, and the side effects weren’t too bad.”* (Participant 1; female)
18	“I don’t know whether, then, you’d prescribe medication type thing or whether you just leave it alone and see what happens, but be mindful that you could have symptomatic, sort of, issues going forward. And if you had those, then you’d probably want to report it, you know, to make doctors more aware, and maybe you’d be more mindful of perhaps your condition and be aware of changes in your body, perhaps.” (Participant 6; male)
19	*“So there obviously are side effects to some of the drugs, so, I guess, unless you were probably showing symptoms, you probably wouldn’t want to go on those sorts of things. But other things like, just, you know, for your bones or whatever, I guess there you would be more than, you know, happier to take that sort of thing in the early stages.”* (Participant 2; female)
20	*“If I’m taking it I’d expect it to work for me. There’s no point in taking anything that is just not going to work for you and it’s not doing the job. Like I’ve always said, I’m not a keen person on medication but if I’m taking it then I would have expectations for it to work.”* (Participant 13; female)

### Modifying risk through lifestyle intervention

Many participants expressed positive views about undertaking lifestyle changes to reduce their risk of developing RA in the future (Table 4, Quote 1 (T4Q1)) or to delay the start of the disease (T4Q2). The main lifestyle factors identified by participants included healthy eating, increasing levels of exercise and smoking cessation. Some participants indicated that they were already trying to live as healthily as possible (although not necessarily focused on preventing RA), but that they would be willing to make additional changes (T4Q3–4). Diet in particular was highlighted as a modifiable risk factor for development of RA and one which patients could take control of (T4Q5). Some participants felt that being overweight and ageing were risk factors for the development of RA (T4Q6).

Participants felt it would be useful to know their personal risk of developing RA so they could modify their lifestyle as a preventive measure (T4Q7). Many participants indicated that they would need information about if and how certain factors such as exercise and diet might impact on the risk of developing RA before deciding what they would do (T4Q8–9).

Some participants expressed more negative views and indicated that they might not be prepared to make lifestyle changes as a preventive measure. Some were more concerned about the perceived negative consequences of making such changes and as a result of that might not want to engage with them (T4Q10). One person indicated that if the only information they have, is that they are at a heightened risk of RA they would not make a change to their diet (T4Q11).

Only a small proportion of the first-degree relatives were current smokers (five, one of whom smoked very rarely) and 2 were ex-smokers. Whereas one smoker described their willingness to give up smoking if a test result identified him as being a person at risk (T4Q12–13), another smoker indicated that a 50% baseline risk would not be enough to consider stopping (T4Q14). A third smoker indicated that they were only prepared to give up smoking if it was confirmed to them that this would definitely prevent them from developing RA (T4Q15). Participants who were not current smokers either identified or accepted that smoking is a risk factor for the development of RA and contemplated how smokers may react negatively to risk information (T4Q16). Another participant pointed out that it can be difficult to avoid passive smoking (T4Q17) and hence the exposure to this risk factor.

### Willingness to take preventive medicines to modify risk

When discussing the possibility of taking preventive medicines if identified as being at high risk of developing RA, many participants highlighted that they would be worried about possible side effects of these medications (T5Q1). They indicated that they would need to think very carefully about the pros and cons of taking preventive medication before making a decision (T5Q2–3). Some participants further worried about the effect of the preventive medication on their existing medical conditions and the potential interaction with current medication (T5Q4–5). Other participants considered the long-term implications of taking medicines, and whether it would, for example, have an impact on family planning in the future (T5Q6). The immunosuppressive properties of disease modifying anti-rheumatic drugs were a particular concern for participants, especially those who had seen the side-effects of such medicines (e.g. an increased frequency of infections) experienced by their family members with RA (T5Q7).

For some participants, weighing up the side-effects of preventive treatment, which were often described as if they were highly likely or certain to occur, against the relative uncertainty of developing RA in the future, meant that they would most likely decline to have preventive medication unless it was shown to be highly effective (T5Q8). Many participants indicated that they would not be willing to take a preventive medication based on a “*probability*” provided by a predictive test and that they would need more “*definitive*” evidence (T5Q9). It was felt that this type of probability information (E.g. ‘You have a 50-50 chance to develop RA’) was too uncertain to inform a choice about medication. Participants reported that a preventive pharmacological therapy would be considered only if the medicine was highly effective in reducing their risk (T5Q10). There were further worries about potentially taking medication for the rest of their lives and the potential consequences of stopping medication (T5Q11). Participants suggested that if a test showed that there was a 50% risk that they would develop RA, this would not be enough for them to accept preventive medication, though they felt that this information might encourage them to monitor for the onset of potential symptoms of RA. Others suggested a relatively high predicted baseline risk might be high enough to convince them to accept preventive medication (e.g. one interviewee suggested this would be a 70–80% risk that they would develop RA; T5Q12).

In addition, several participants expressed negative opinions about taking medicines in general, in some cases as a result of past experiences with being prescribed medication that they perceived to be unnecessary,-and they anticipated that this attitude would stop them from taking medication to prevent the onset of RA (T5Q13–14). Others indicated that, based on the assumption of an equal level of risk reduction, they would rather prefer to make lifestyle changes as opposed to taking long term medication (T5Q15) in order to reduce their risk of developing RA.

There was also a group of participants who could understand why preventive medicine may be prescribed if they were found to be at risk of developing RA in the future and anticipated that they would be willing to accept such treatment (T5Q16). This was especially the case for those participants who had experienced the negative impact of the illness on their relatives (T5Q17).

Participants were supportive of the idea that predictive testing could be useful to alert those who are at high risk to be vigilant for the early symptoms of RA, so that treatment could be initiated as soon as symptoms appeared (T5Q18). Many participants suggested that they would prefer this strategy of early intervention during the symptomatic phase as opposed to taking a preventive medicine prior to the onset of symptoms (T5Q19).

Finally, for some participants the issue of engaging with a preventive intervention depended on an expectation that it would definitely be effective (T5Q20).

## Discussion

Given the current impetus to identify early intervention points in the RA disease pathway, it is important to understand the perspectives of those at increased risk of developing RA about possible preventive interventions. The current study looked at one particular at risk group, first-degree relatives, and found that many anticipated being happy to make lifestyle changes such as losing weight, increasing exercise and changing diet to modify their risk of developing RA. However, there was less enthusiasm about a pharmacological approach, at either the pre-symptomatic or the symptomatic at risk stage [9, 24].

The interviewees’ reactions to the possibility of making lifestyle changes might be in part due to the social desirability of giving such a response and it is unclear how many would actually adopt these behaviours. One would expect that using well trained interviewers who, with one exception were non-Rheumatologists, went some way to avoiding such responses. Further, the results from the trial conducted by Sparks and colleagues [21] indicate that first-degree relatives were both motivated to modify their behaviour and reported making actual lifestyle changes (e.g. increasing their fish intake; quitting smoking) especially after receiving personalised information about their risk status and the impact of health behaviours on their risk status. This implies that first-degree relatives might indeed follow through on making lifestyle changes after receiving relevant (and personalised) information. Indeed, some interviewees in the current study indicated that they would appreciate further information about the impact of such behavioural changes on their risk of developing RA. Others said that they would require definitive confirmation that it would lead to a significant reduction in their risk of developing RA before contemplating making such changes. Interviewees were more reluctant to contemplate making changes in their lifestyle if they considered there to be negative effects of making such changes. Stopping smoking can be a difficult lifestyle change to make due to the addictive nature of tobacco [37]. In our sample, the perceived benefits of stopping smoking in relation to RA would make some participants more likely to consider smoking cessation if they were found to be at risk of developing RA. Others would need more certainty that this lifestyle change would eliminate their risk of developing RA altogether. These findings are in line with research in other at risk groups for chronic conditions such as diabetes or cardiovascular disease (CVD). In a study where individuals were given their actual risk score for CVD, some indicated that they had made or intended to make lifestyle changes such as diet change or stopping smoking, whereas others would not make those changes, for example, because they disbelieved the risk score or downplayed their risk, or because they actively resisted making such changes and for example ‘could not be bothered’ to stop smoking [38]. A person’s motivation to be healthy prior to being tested for a chronic condition, will impact on how they perceive their risk and their willingness to make behavioural changes once they receive their results [32]. Further research is needed to comprehensively assess predictors of responses to information about disease risk.

The finding that first-degree relatives were more hesitant about preventive medication is in agreement with how patients with RA in previous research felt that their family members might react if given the option of preventive medicine [30]. In line with findings from previous research in RA (e.g [27, 39]), potential side effects, long-term implications of taking the medication and possible interactions with existing conditions and medications were a major concern for many of our interviewees and they indicated that they would weigh up perceived benefits and potential harms before making a decision about therapy. Personal experiences of RA in family members influenced decision making about the value of preventive medication for several interviewees. On the one hand, those who had observed the side effects of medications such as Disease Modifying Anti-Rheumatic Drugs (DMARDs) experienced by their relatives indicated that they might be more reluctant to take the medication as a preventive measure. On the other hand, some interviewees, who had witnessed the negative effects of RA on their relative’s daily life, were keen to use preventive medicine to reduce the risk of developing musculoskeletal symptoms. Acceptance of preventive medication was further associated with the issue of certainty of future RA development. Many felt that uncertainty around future development of RA would discourage them from accepting medications, which they associated with negative side effects. Some interviewees indicated that they would only consider taking medication after the onset of symptoms, and the main value of predictive screening would be to identify the extent to which they should be on the alert for the start of RA symptoms – at which point they would consider therapy which is also in line with findings from a recent focus group study with first-degree relatives [39]. Interviewees in the current study were further not directly asked what they perceived their current risk of developing RA to be, but it is likely that different levels of perceived risk might have had an impact on their views on preventative measures. Future research should explore this relationship further.

Interviewees in this study were not provided with detailed information about the likely duration, frequency of method of administration of preventive treatments. Some of our interviewees appeared to assume that treatment to reduce their risk of RA would involve long-term medication and associated extended risk of side effects. This could account for negative viewpoints towards such treatment. However the trial that is currently evaluating preventive therapy (hydroxychloroquine) for this group (asymptomatic individuals) involves a 12 month course [25], which could be considered as relatively long term treatment.

A recent discrete choice experiment looking at first-degree relatives’ preferences related to pharmacological interventions, suggested that method of administration may be an important determinant of preventive treatment acceptability [40]. In contrast a related best worst scaling pilot study found that the efficacy and risks of treatment were more important than method of administration in decision making about preventive treatments [24]. Further quantitative evidence is needed to clarify the relative importance of treatment related and other attributes in the choices made by at risk individuals about preventive therapy for RA.

Collectively these findings suggest that educational approaches will be needed to support preventive strategies, and should include information about the benefits and risks of lifestyle changes and pharmacological interventions as well as details about the nature of the intervention itself. This supportive information should be given in the context of personalised risk estimation with clear (and where possible quantitative) information about the potential for risk reduction associated with such interventions [41]. As predictive algorithms for RA continue to evolve and improve, the interplay between genetic and environmental factors could be highlighted and it is possible for interactive tools to give people personalised information about their risk of developing RA given their genetic make-up and current lifestyle, demonstrating how RA risk can be changed by making certain lifestyle changes. The recent trial of such a tool shows a positive impact on both intended and actual risk related behaviour change [21]. However, there remains a misalignment between public expectations of risk assessment (e.g. being able to predict with 100% certainty whether or not an individual will develop a disease) and the realistic possibilities of predictive models – which provide probabilistic risk estimates with varying confidence intervals [30]. Levels of confidence in estimates of risk or efficacy may impact on preferences for preventive treatments [30, 40, 42], therefore informational resources accompanying preventive interventions should therefore also manage expectations around the accuracy of risk information [42].

Our results further highlight the need to communicate risk information in a way that is sensitive to the personal/family context or life situation of each individual. One could also provide information that is specifically tailored for different groups with varying experiences of RA in order to provide a richer, more personalised reflection of their own experience. First-degree relatives are a distinct at risk group and their views are likely to be influenced by their personal experiences with RA. As such, their information needs are likely to be different from those of the general population and of other at risk groups, such as patients with clinically suspect arthralgia [43] or undifferentiated arthritis. In ongoing research we are exploring the perspectives of other at risk groups and the wider public, and predictors of perceptual variability within each of these groups.

A number of limitations need to be considered when interpreting the findings of the current research. Firstly, the proportion of men in the sample was relatively small. Although this does reflect the fact that fewer men than women develop RA [1], important viewpoints may have been missed in the current study and future research should aim to include a larger sample size of men. Similarly, although we interviewed relatives in three different European countries, the current sample is not ethnically diverse and as such does not comprehensively represent the population of each of the participating countries. The sample size and qualitative approach used here do not allow for comparative analysis between the three countries and assessment of cross-cultural differences are not possible on the basis of the current data. Future research should therefore specifically address these omissions in order for screening and intervention programmes to be tailored for as wide a target population as possible and to provide related information in a way that is gender or culturally appropriate. Further quantitative evidence is needed to explore psychosocial and cultural predictors of preference heterogeneity and the degree to which at risk individuals trade off positive and negative aspects of preventive interventions for RA.

Since access to first-degree relatives is usually indirectly through patients with existing RA, future research needs to extend and quantify findings of previous research [30] related to if and how RA patients are likely to communicate with their first-degree relatives about their increased risk of developing RA and risk reduction options. This information will help to facilitate the development of efficient preventive approaches and recruitment of participants to preventive studies. The current research discussed participants’ willingness to make (hypothetical) lifestyle changes. However, although there is consensus about some lifestyle factors which influence a person’s personal risk of developing RA (such as smoking), more research is needed to understand the degree of risk reduction, in the context of different genetic backgrounds, associated with risk related lifestyle changes [21]. Only when such data are available can those at risk make informed decisions about preventive intervention.

## Conclusion

The current research gives an indication of first-degree relatives’ perceptions of lifestyle changes and preventive medication to reduce their risk of developing RA and the factors associated with acceptability of such preventive measures. It identifies factors which should be highlighted in informational materials aimed at first-degree relatives who are considering participating in related research studies or preventive approaches as well as highlighting factors which warrant further investigation.

## Abbreviations

CVD: Cardiovascular Disease
DMARDs: Disease Modifying Anti-Rheumatic Drugs
RA: Rheumatoid Arthritis
